# A cross-cultural comparison between South African and British students on the Wechsler Adult Intelligence Scales Third Edition (WAIS-III)

**DOI:** 10.3389/fpsyg.2015.00297

**Published:** 2015-03-13

**Authors:** Kate Cockcroft, Tracy Alloway, Evan Copello, Robyn Milligan

**Affiliations:** ^1^Department of Psychology, University of the Witwatersrand, JohannesburgSouth Africa; ^2^Psychology, University of North Florida, Jacksonville, FLUSA

**Keywords:** cross-cultural, culture-fair, intelligence tests, WAIS-III, working memory

## Abstract

There is debate regarding the appropriate use of Western cognitive measures with individuals from very diverse backgrounds to that of the norm population. Given the dated research in this area and the considerable socio-economic changes that South Africa has witnessed over the past 20 years, this paper reports on the use of the Wechsler Adult Intelligence Scale Third Edition (WAIS-III), the most commonly used measure of intelligence, with an English second language, multilingual, low socio-economic group of black, South African university students. Their performance on the WAIS-III was compared to that of a predominantly white, British, monolingual, higher socio-economic group. A multi-group confirmatory factor analysis showed that the WAIS-III lacks measurement invariance between the two groups, suggesting that it may be tapping different constructs in each group. The UK group significantly outperformed the SA group on the knowledge-based verbal, and some non-verbal subtests, while the SA group performed significantly better on measures of Processing Speed (PS). The groups did not differ significantly on the Matrix Reasoning subtest and on those working memory subtests with minimal reliance on language, which appear to be the least culturally biased. Group differences were investigated further in a set of principal components analyses, which revealed that the WAIS-III scores loaded differently for the UK and SA groups. While the SA group appeared to treat the PS subtests differently to those measuring perceptual organization and non-verbal reasoning, the UK group seemed to approach all of these subtests similarly. These results have important implications for the cognitive assessment of individuals from culturally, linguistically, and socio-economically diverse circumstances.

## Introduction

Intelligence (IQ) testing is widely used in cross-cultural contexts, yet there are still persistent critiques that such tests can perpetuate racist beliefs and lead to discrimination (Helms, 1992; Kaufman, 1994). Although it has been suggested that non-verbal, visuospatial IQ subtests may be free of cultural and linguistic biases, this assumption has been contested (Rosselli and Ardila, 2003). Normative data for most tests of cognitive ability are predominantly based on monolingual, reasonably aﬄuent, English first language individuals and these norms are still routinely applied to culturally and linguistically diverse individuals. In instances where other cultural groups are represented in the standardization samples of tests developed in Europe and the United States (US), they tend not to include cultures beyond these continents. This makes the application of such measures to individuals from culturally, linguistically and socio-economically different circumstances problematic.

This matter has particular relevance in South Africa, a country with 11 official languages, a majority of English second language speakers, and high levels of poverty. South Africa (SA) shares historical and economic ties with the United Kingdom (UK) and is part of the Commonwealth of Nations, yet represents a unique blend of linguistic and cultural diversity. For those South African cultures (white English- and Afrikaans-speaking) that share many similarities with Western Europe, IQ performance tends to be comparable (Shuttleworth-Edwards, 1996; Claassen et al., 2001). However, the majority of the SA population belongs to cultures, and linguistic and socio-economic circumstances that are very different from that of Western Europe and the typical IQ test normative groups.

Despite this, the Wechsler Adult Intelligence Scale Third Edition (WAIS-III) has been adapted and standardized for use with all South Africans. This adaptation has been criticized for failing to re-pilot the adaptations for possible cultural and language biases and for neglecting to stratify the norms according to quality of education (Shuttleworth-Edwards et al., 2004; Foxcroft and Aston, 2006). The reason for the latter critique is that the current SA educational systems (former Model C, ex-DET, and privately funded) offer education that differs vastly in quality. Former Model C state schools are those that were previously reserved for the education of white children under apartheid and were modeled on UK public schools. They are comparable to privately funded schools and generally provide a high standard of education. In contrast, schooling provided by the legacy Department of Education and Training (DET) for black children under apartheid, continues to be constrained by limited resources and large classes despite new educational policies that mandate fairer allocation of resources. Although many black children now attend the former Model C and private schools, a large proportion have no option but the ex-DET schools (Shuttleworth-Edwards et al., 2004; Fleisch, 2008).

An advantaged, Western-style schooling teaches problem solving, as well as test-taking skills, which are heavily drawn on in traditional IQ tests (Nell, 2000; Shuttleworth-Edwards et al., 2013). Shuttleworth-Edwards et al. (2004) found that both Verbal and Performance IQs on the WAIS-III were negatively affected by a poor quality education in a sample of black Africans with an African language as their mother-tongue. Consequently, these authors argue for the stratification of IQ test norms in terms of both level and quality of education to allow for some control over the considerable differences between educational systems. Level and quality of education have been shown to correlate significantly with performance on most WAIS-III indices and subtests (Heaton et al., 2003) in European (Kessels and Wingbermuhle, 2001; Grégoire, 2004), American (Razani et al., 2006), Australian (WAIS-R, Shores and Carstairs, 2000), and African samples (Shuttleworth-Edwards et al., 2004). Quality of education appears to be a more important variable than years of education, and may explain some of the discrepant findings in cross-cultural comparisons of the WAIS-III that have emerged despite careful matching for educational level (Manly et al., 2004; Shuttleworth-Edwards et al., 2004).

In addition to level and quality of education, past studies have identified cultural, linguistic, and socio-economic factors as affecting IQ test performance (Harris et al., 2003; Turkheimer et al., 2003; Manly et al., 2004; Shuttleworth-Edwards et al., 2004; Razani et al., 2006; Boone et al., 2007; Walker et al., 2009). In considering the effects of these complex and inter-related variables on IQ test performance, we have focused our review on empirical evidence concerning the WAIS-III, its abbreviated form, the WASI-III and its predecessor, the WAIS-R (Revised), and not on other versions of this test which are structurally quite different, or on evidence from children’s IQ tests, which is complicated by developmental considerations.

The influence of cultural background on IQ test performance has received limited attention in the last two decades and consequently many of the available studies are dated. Most of the studies reviewed here are based on the assumption that cultural or ethnic groups reflect a homogenous set of socio-cultural characteristics, which is not necessarily the case, and which makes this field particularly difficult to investigate. A summary of the most recent available studies shows that African Americans tend to score significantly lower than white and Hispanic groups on the WAIS-III Verbal Comprehension (VCI), Perceptual Organization (POI), and Processing Speed (PSI) indices (Heaton et al., 2003), on the WAIS-R subtests (Kaufman et al., 1991) and WAIS-R Full Scale IQ (Byrd et al., 2006). Other comparisons found that African American individuals score lower than Caucasian individuals on WAIS-III Digit Span, but not Digit Symbol (Razani et al., 2007), while individuals of Hispanic, Asian, and Middle Eastern backgrounds score lower than those of English-speaking backgrounds on WASI-III Vocabulary and Similarities (Razani et al., 2006). The latter study found no cross-cultural effects on Block Designs and Matrix Reasoning, although these subtests have been widely criticized elsewhere as lacking cross-cultural validity (Kaufman et al., 1988; Marcopulos et al., 1997; Dugbartey et al., 1999; Shuttleworth-Edwards et al., 2004).

The most comprehensive cross-cultural studies on the WAIS-III have been conducted by Shuttleworth-Edwards (1996), Shuttleworth-Edwards et al. (2004, 2013), with black and white Southern Africans. They found that scores for black and white Southern Africans with an advantaged education were comparable to those in the US WAIS-III standardization, while black Africans with disadvantaged educational backgrounds scored up to 20 IQ points lower (Shuttleworth-Edwards et al., 2004). Further refinement of the latter study by removing non-South Africans from the data set and replacing them with data from South African, Xhosa-speaking participants, revealed concomitant increases in WAIS-III subtest, Index and IQ scores as the quality of education increased (Shuttleworth-Edwards et al., 2013). These authors also found that the subtests which revealed the most cross-cultural differences (irrespective of quality of education) were Object Assembly, Symbol Search, Picture Arrangement and Block Design, demonstrating that both verbal and non-verbal subtests are susceptible to such influences.

Systematic comparisons between the various cross-cultural studies on IQ test performance are difficult to undertake due to methodological differences, for example not all of the WAIS-III subtests, Index and IQ scores are consistently reported, while several studies did not match comparison groups for education. While it has been suggested that processing based measures which draw on fluid problem solving skills are likely to be more culture fair than those that rely on acquired, long-term learning (Campbell et al., 1997; Nell, 2000), the review of the literature indicates that there does not appear to be a single WAIS-III subtest that consistently showed no cross-cultural effects, and the most consistent of these effects appear in the verbal subtests.

Effects of culture are likely to be most pronounced where cultural background is most divergent from an English first language, Western culture (Ardila and Moreno, 2001). In support of this, higher performance on IQ tests has been demonstrated by individuals from Mexican–American backgrounds who possess greater ‘Anglo socio-cultural characteristics’ (Gonzales and Roll, 1985; Razani et al., 2006, p. 777). Among the English second language speakers of the WAIS-III standardization sample, Harris et al. (2003) found that acculturation variables such as language preference, years of residence in the US and length of education in the US all accounted for significant variance in VCI and PSI Indices. Degree of acculturation also accounted for the lower scores of African American individuals on the WAIS-R Block Design (Manly et al., 1998). Degree of acculturation has been shown to account for significant variability in the PSI of the WAIS-III (Harris et al., 2003; Kennepohl et al., 2004). While there is some variation across studies in terms of acculturation variables, most include language usage, test-wiseness (test-taking skill, motivation, and perceptions of test face validity), socio-economic status (SES), home and school environments, and level and quality of education (Kennepohl et al., 2004; Shuttleworth-Edwards et al., 2004; Perry et al., 2008). An aspect of acculturation, test-wiseness, has been reported as ‘the most powerful moderator of test performance,’ exerting strong effects on both verbal and non-verbal test performance (Nell, 2000, p. 133). Extent of acculturation will also influence how a particular construct being measured in an IQ subtest is perceived in the target population. In this regard, few studies have considered and statistically evaluated the presence of measurement invariance between comparison groups. Violations of measurement invariance would imply that the same construct is not being measured across different cultural groups and could complicate meaningful interpretation of test data (Dolan et al., 2006; Milfont and Fischer, 2010).

It is evident that performance on IQ tests is influenced by a range of socio-cultural variables which may lead to considerable heterogeneity in performance, particularly when the testees come from backgrounds that are very different to that of the norm sample. However, the body of research in this area is at least 10 years-old. Flynn effects, which refer to an average increase of three IQ points per decade (with two IQ points increase per decade on the Verbal Scale and four IQ points on the Performance or non-verbal Scale) have been reported in many nations (Barber, 2005; Flynn, 2007, 2009). To the authors’ knowledge, there are no published data on Flynn effects in black South Africans (the sample of interest in the current study), but data from white and Indian South Africans (te Nijenhuis et al., 2011) show increases in the expected direction and magnitude. These effects are predominantly driven by environmental factors (te Nijenhuis, 2013). Thus, the considerable socio-economic transformations that South Africa has experienced over the last 20 years could mean that the dated cross-cultural research may no longer be relevant to the new generation of young adult South Africans born after the end of apartheid. As an initial attempt to explore this possibility, the purpose of this study was to compare performance on the WAIS-III between two diverse groups, namely a non-standard and a standard population of university students. The latter group was similar to the UK WAIS-III normative group, namely predominantly white, monolingual English speakers from mid-level SES backgrounds in Britain. The non-standard comparison group were multilingual, black African, English second language speakers from low socio-economic circumstances in South Africa. No prior hypotheses were stipulated, rather we were interested in whether there would be any differences between the groups on the various subtests of the WAIS-III.

## Materials and Methods

### Participants

#### British Sample

There were 349 undergraduate UK university students (33% male), ranging from 18 to 58 years (*M* = 27.18 years; SD = 8.74 years). All were monolingual English speakers, from middle-class backgrounds. SES was determined from university reports on the backgrounds of students. Fifty-two percent resided in urban areas and 33.4% in accessible rural areas. The ethnic distribution of this group (71% white, 1% black, and 18% other, i.e., Asian, Indian, and international students) was broadly similar to that of the UK WAIS-III norm sample (94% white, 3.36% black, and 2.44% other; Wechsler, 1997). Ten percent of the UK sample did not indicate an ethnic group on their biographical questionnaire. All participants had received primary and secondary schooling in English, at public and private schools.

#### South African Sample

These were 107 undergraduate black African students enrolled at an English medium University (40% male*; M* = 20.79 years, SD = 1.56 years; range: 18–25 years). Since the quality of primary and secondary education ranges vastly in post-apartheid South Africa and has been shown to affect performance on cognitive tests (Shuttleworth-Edwards et al., 2013), students who had attended schools in the lowest three quintiles of the South African government school system, which represent the poorest schools, were recruited through advertisements on the university campus (Department of Education, 2008). The majority (82%) of the SA participants underwent primary and secondary schooling in English. All were multilingual and spoke, on average, four languages (English and three African languages). In all cases, an African language was the first language, and for 65% English was the L2. Fifty-five percent had acquired English before the age of six and the remainder before 13.

All of the SA participants came from low socio-economic circumstances. The majority (82%) resided in rural areas, in a basic brick house with running water and electricity. Hardly any (98%) had washing machines, microwave ovens, or tumble-dryers. Less than 1% of families owned a motor vehicle or personal computer. Two-thirds (67%) had attended preschool; 52% came from single parent homes, and 28% never knew their fathers. Forty-two percent of mothers had not completed high school, and 35% had post-secondary school qualifications.

### Materials and Procedure

An established measure of intelligence, the WAIS-III (Wechsler, 1997), was administered. The South African item administration (Claassen et al., 2001) was used with the SA sample. This version is similar to the UK version, with minor alterations to the content to make it more appropriate to a SA context, such as the use of ‘rands’ instead of ‘pounds,’ and ‘cool drinks’ instead of ‘soft drinks’ in the Arithmetic subtest. In addition, the SA standardization resulted in amendments to four items in the Vocabulary subtest, five in the Information subtest and two in the Comprehension subtest to make them more appropriate for SA testees. Claassen et al. (2001), in their standardization of the WAIS-III for South Africans, found that the differences in scaled scores between individuals tested with both the original UK items and the altered SA items were very small, and thus it is unlikely that this would have exerted any significant effect on the comparisons in the current study.

The WAIS-III comprises 14 subtests, of which 13 were administered (Object Assembly, which does not contribute to the Full Scale IQ and is an optional subtests, was not administered). In the *Vocabulary* subtest, the individual has to produce a definition of a given word. In the *Similarities* subtest, the individual has to describe how two words are similar. In the *Information* subtest, the individual is asked general knowledge questions. These subtests are combined to create the VCI. The *Comprehension* subtest is not part of the VCI, but contributes to the Verbal IQ. In this subtest, the individual provides a response to various social situations. The following subtests comprise the POI. In *Block Design*, the individual assembles red and white blocks to recreate a given shape. *Matrix Reasoning* consists of a sequence of designs where the individual fills in a missing design piece from a selection of options. In *Picture Completion,* the individual is required to recognize the missing part in a set of pictures. *Picture Arrangement* is not part of the POI, but contributes to the Performance IQ. It requires the individual to arrange pictures in a logical sequence. The PSI comprises *Coding*, where symbols are matched with numbers under a time constraint; and *Symbol Search*, where the individual has to rapidly scan a set of symbols to identify a target. The Working Memory Index (WMI) comprises three subtests: *Digit Span*, where the individual has to recall numbers in forward and backward order; *Letter–Number Sequencing (LNS)*, where the individual recalls a string of given letters and numbers in numerical and then alphabetical order; *Arithmetic,* where the individual responds to mental math questions.

Testing was carried out in a one-to-one, single session on the campus, by two psychologists trained in the administration of the WAIS-III (one for the UK sample and one for the SA sample). Both SA and UK protocols were scored according to the UK WAIS-III manual scoring criteria, with the exception of the few changed content items on the SA administration, in which cases the SA scoring had to be used. Subtest scale scores, Full Scale IQ, Verbal IQ, Performance IQ and Factor Indices were calculated using the UK norms for both the SA and UK samples (Wechsler, 1997). All protocols were scored twice, once by the psychologists who administered the WAIS-III, and once by the primary (SA protocols) and secondary (UK protocols) authors, who were both trained and experienced in WAIS-III administration.

The study was approved by the ethics committees of the University of the Witwatersrand, Johannesburg and the University of Stirling, UK. Informed, written consent was obtained from all participants with appropriate opportunities for withdrawal without prejudice.

## Results

### Descriptive Statistics and Correlation Analyses

Descriptive statistics for the WAIS-III subtests, Index and IQ scores are provided in **Table 1**.

**Table 1:** Descriptive statistics of scores for all IQ subtests (standard scores unless otherwise stated).

	South Africa		UK		*Post hoc* (*F)*	Cohen’s *d*
	*M*	SD	*M*	SD		
Similarities^∗^	8.12	1.40	14.22	3.04	437.69^∗∗^	2.58
Vocabulary^∗^	8.92	2.02	14.56	2.66	462.65^∗∗^	2.39
Information^∗^	10.19	3.21	11.75	2.72	32.57^∗∗^	0.52
Comprehension^∗^	8.99	2.50	12.48	2.59	163.36^∗∗^	1.37
Verbal Comprehension Index	94.81	10.25	120.12	14.84	311.68^∗∗^	1.98
Block Design^∗^	8.67	2.61	10.66	2.87	44.29^∗∗^	0.73
Picture Arrangement^∗^	8.33	2.82	10.08	2.26	43.486^∗∗^	0.68
Matrix Reasoning^∗^	10.68	2.37	10.59	2.71	0.10	0.04
Picture Completion^∗^	8.05	2.11	10.57	3.13	67.93^∗∗^	0.94
Perceptual Organization Index	94.28	10.94	103.09	14.69	40.57^∗∗^	0.68
Digit Symbol-Coding^∗^	9.75	2.51	7.72	2.68	49.40^∗∗^	0.78
Symbol Search^∗^	9.71	2.67	8.25	2.62	25.00^∗∗^	0.55
Processing Speed Index	98.18	12.27	88.99	13.20	41.51^∗∗^	0.72
Digit Span^∗^	9.35	2.26	9.50	2.94	0.20	0.06
Letter–Number Sequence^∗^	9.17	2.42	8.88	2.72	2.64	0.11
Arithmetic^∗^	8.66	2.53	9.58	3.14	0.17	0.32
Working Memory Index	93.83	11.40	95.45	14.09	<1.00	0.13
Verbal IQ	93.63	9.96	112.14	13.92		1.53
Performance IQ	93.56	10.05	98.89	13.94		0.44
Full Scale IQ Index	93.27	8.94	106.95	12.90		1.23

^∗^Scale scores (*M* = 10, SD = 3); ^∗∗^*p* < 0.001.

Pearson’s correlation coefficients between the WAIS-III subtests are reported for each sample in **Table 2**. As expected, the subtests within each Index (VCI, WMI, POI, and PSI) were significantly related to one another (*r*’s ranging from 0.20 to 0.77) for both samples.

**Table 2:** Correlations between WAIS-III subtests, for UK and SA samples.

Measures	1	2	3	4	5	6	7	8	9	10	11	12	13	14	15	16
1. V	1	0.71^∗∗^	0.32^∗∗^	0.10	0.65^∗∗^	0.61^∗∗^	0.17^∗∗^	0.23^∗∗^	0.05	0.32^∗∗^	0.31^∗∗^	0.28^∗∗^	0.15^∗∗^	0.72^∗∗^	0.30^∗∗^	0.62^∗∗^
2. S	0.52^∗∗^	1	0.37^∗∗^	0.14^∗^	0.60^∗∗^	0.65^∗∗^	0.26^∗∗^	0.27^∗∗^	0.05	0.36^∗∗^	0.39^∗∗^	0.29^∗∗^	0.12^∗^	0.75^∗∗^	0.34^∗∗^	0.66^∗∗^
3. A	0.43^∗∗^	0.41^∗∗^	1	0.38^∗∗^	0.35^∗∗^	0.51^∗∗^	0.46^∗∗^	0.34^∗∗^	0.18^∗∗^	0.46^∗∗^	0.48^∗∗^	0.42^∗∗^	0.28^∗∗^	0.67^∗∗^	0.47^∗∗^	0.67^∗∗^
4. DS	0.21^∗^	0.18	0.32^∗∗^	1	0.14^∗^	0.45^∗∗^	0.61^∗∗^	0.17^∗∗^	0.26^∗∗^	0.32^∗∗^	0.25^∗∗^	0.33^∗∗^	0.36^∗∗^	0.52^∗∗^	0.34^∗∗^	0.51^∗∗^
5. I	0.62^∗∗^	0.56^∗∗^	0.52^∗∗^	0.15	1	0.61^∗∗^	0.22^∗∗^	0.27^∗∗^	0.05	0.29^∗∗^	0.35^∗∗^	0.31^∗∗^	0.12^∗^	0.71^∗∗^	0.33^∗∗^	0.63^∗∗^
6. C	0.60^∗∗^	0.53^∗∗^	0.41^∗∗^	0.31^∗∗^	0.56^∗∗^	1	0.38^∗∗^	0.25^∗∗^	0.08	0.41^∗∗^	0.38^∗∗^	0.38^∗∗^	0.23^∗∗^	0.85^∗∗^	0.36^∗∗^	0.74^∗∗^
7. LNS	0.23^∗^	0.20^∗^	0.39^∗∗^	0.45^∗∗^	0.18	0.19^∗^	1	0.24^∗∗^	0.24^∗∗^	0.36^∗∗^	0.35^∗∗^	0.39^∗∗^	0.31^∗∗^	0.49^∗∗^	0.39^∗∗^	0.51^∗∗^
8. PC	0.19^∗^	0.25^∗^	0.20^∗^	0.09	0.25^∗^	0.19^∗^	0.19^∗^	1	0.26^∗∗^	0.44^∗∗^	0.49^∗∗^	0.65^∗∗^	0.33^∗∗^	0.35^∗∗^	0.74^∗∗^	0.61^∗∗^
9. DSC	0.09	0.16	0.13	0.16	0.03	–0.04	0.20^∗^	0.08	1	0.33^∗∗^	0.30^∗∗^	0.50^∗∗^	0.77^∗∗^	0.17^∗∗^	0.61^∗∗^	0.42^∗∗^
10. BD	0.20^∗^	0.28^∗∗^	0.34^∗∗^	0.36^∗∗^	0.33^∗∗^	0.21^∗^	0.24^∗^	0.42^∗∗^	0.28^∗∗^	1	0.55^∗∗^	0.66^∗∗^	0.43^∗∗^	0.48^∗∗^	0.76^∗∗^	0.72^∗∗^
11. MR	0.06	0.07	0.21^∗^	0.19^∗^	0.09	0.17	0.17	0.39^∗∗^	0.07	0.40^∗∗^	1	0.69^∗∗^	0.39^∗∗^	0.49^∗∗^	0.75^∗∗^	0.73^∗∗^
12. PA	0.24^∗^	0.14	0.31^∗∗^	0.14	0.35^∗∗^	0.26^∗∗^	0.10	0.30^∗∗^	0.12	0.26^∗∗^	0.21^∗^	1	0.54^∗∗^	0.47^∗∗^	0.86^∗∗^	0.77^∗∗^
13. SS	0.09	0.12	0.18	0.22^∗^	0.06	0.06	0.25^∗^	0.24^∗^	0.45^∗∗^	0.39^∗∗^	0.25^∗∗^	0.19^∗^	1	0.30^∗∗^	0.64^∗∗^	0.52^∗∗^
14. VIQ	0.77^∗∗^	0.69^∗∗^	0.73^∗∗^	0.50^∗∗^	0.80^∗∗^	0.80^∗∗^	0.39^∗∗^	0.27^∗∗^	0.09	0.40^∗∗^	0.20^∗^	0.34^∗∗^	0.16	1	0.49^∗∗^	0.87^∗∗^
15. PIQ	0.23^∗^	0.28^∗∗^	0.37^∗∗^	0.31^∗∗^	0.32^∗∗^	0.22^∗^	0.31^∗∗^	0.66^∗∗^	0.50^∗∗^	0.75^∗∗^	0.62^∗∗^	0.61^∗∗^	0.48^∗∗^	0.40^∗∗^	1	0.82^∗∗^
16. FSIQ	0.64^∗∗^	0.61^∗∗^	0.67^∗∗^	0.49^∗∗^	0.71^∗∗^	0.66^∗∗^	0.41^∗∗^	0.52^∗∗^	0.32^∗∗^	0.65^∗∗^	0.46^∗∗^	0.55^∗∗^	0.35^∗∗^	0.88^∗∗^	0.78^∗∗^	1

SA sample below the diagonal, UK sample above the diagonal; ^∗^*p* < 0.05; ^∗∗^*p* < 0.001; V, Vocabulary; S, Similarities; A, Arithmetic; DS, Digit Span; I, Information; C, Comprehension, LNS, Letter–Number Sequencing; PC, Picture Completion; DSC, Digit-Symbol Coding; BD, Block Design; MR, Matrix Reasoning; PA, Picture Arrangement; SS, Symbol Search; VIQ, Verbal IQ; PIQ, Performance IQ; FSIQ, Full Scale IQ.

### Group Comparisons

A series of multivariate analysis of variances (MANOVAs) were conducted on the age-adjusted WAIS-III measures to investigate potential cultural differences between the UK and SA students (**Table 1**). The first MANOVA was conducted on the WAIS-III Index scores (VC, PO, WM, PS). The overall group term associated with Hotelling’s T-test was significant (*F* = 87.53, *p* < 0.001; $\eta_{p}^{2}$ = 0.44). *Post hoc* pairwise comparisons (*p*< 0.001, Bonferroni adjustment for multiple comparisons) indicated that the UK students scored significantly higher than the South African students in the subtests of the VCIs and POIs. In contrast, the South African students scored significantly higher than the UK students in the PSI. There was no significant group difference in the subtests of the WMI.

Follow-up MANOVAs were conducted on the subtests associated with the IQ indices that yielded significant group differences. The first was performed on the scaled scores of the VCI subtests (Vocabulary, Information, Similarities) and Comprehension. The overall group term associated with Hotelling’s T-test was significant ( *F=* 160.08, *p*< 0.001; $\eta_{p}^{2}$ = 0.59). *Post hoc* pairwise comparisons (*p*< 0.001, Bonferroni adjustment for multiple comparisons) showed that the UK students scored significantly higher than the SA students on all four verbal subtests. This suggests that these subtests draw on culture-specific, acquired knowledge, and appear to be affected by these differences.

The second MANOVA was performed on the scaled scores of the POI subtests (Block Design, Matrix Reasoning, Picture Completion) and Picture Arrangement. The overall group term associated with Hotelling’s T-test was significant (*F =*31.17, *p*< 0.001; $\eta_{p}^{2}$ = 0.22). *Post hoc* pairwise comparisons (*p*< 0.001, Bonferroni adjustment for multiple comparisons) indicated that the UK students performed significantly better on all non-verbal subtests, except for Matrix Reasoning, compared to their SA counterparts.

The last MANOVA was performed on the scaled scores for the PSI subtests (Digit-Symbol Coding and Symbol Search). The overall group term associated with Hotelling’s T-test was significant (*F =*24.23, *p*< 0.001; $\eta_{p}^{2}$ = 0.10). *Post hoc* pairwise comparisons revealed that the SA students achieved significantly higher scores than the UK students on both subtests (*p*< 0.001, Bonferroni adjustment for multiple comparisons).

### Discriminant Function Analysis

In order to identify which measures could uniquely differentiate the two cultural groups, discriminant function analyses were conducted for the nine WAIS-III subtests that produced significant group differences, in a stepwise fashion. Wilks’ Lambda was significant for these subtests (*p*< 0.001): Vocabulary, λ(1,455) = 0.42; Information, λ(1,454) = 0.42; Similarities, λ(1,453) = 0.40; Coding, λ(1,452) = 0.38; Picture Arrangement, λ(1,451) = 0.37; Symbol Search, λ(1,450) = –0.37; Picture Completion, λ(1,448) = 0.36; Comprehension and Block Design did not differentiate between the two cultural groups. Performance on these seven WAIS-III subtests was sufficient to correctly assign group membership for 93% of both the UK and SA students (91% for UK and 99% for SA). This analysis established that higher VC scores typically characterize the UK students, while higher PS scores typify the SA students.

### Multi-Group Confirmatory Factor Analyses

In order to detect potential measurement invariance across groups, a multi-group confirmatory factor analysis (MGCFA) was conducted. This analytic technique has been advocated particularly in the investigation of group differences in IQ test scores (Dolan et al., 2004, 2006; Wicherts and Dolan, 2010). We tested a four-factor model based on the specified Wechsler structure, namely VCI, POI, WMI, and PSI. Group differences for these four factors were investigated by fitting a series of increasingly restrictive models and evaluating the difference in fit between the restricted model and the less-restricted model (see **Table 3**). Step 1 is a model where parameters were freely estimated; in Step 2, the factor loadings were restricted; and in Step 3, we restricted the measurement intercepts to be invariant, while allowing the factor means to differ across groups. As the successive models were nested, we conducted chi-square difference tests (χ^2^) between each Step (Bollen, 1989).

**Table 3:** Goodness of fit statistics and path coefficients for latent constructs (*p* < 0.05) as a function of country.

Country	χ^2^ (df)	CFI	RMSEA	AIC	SRMR	VC-PO F1-F2	VC-WM F1-F3	VC-PS F1-F4	PO-WM F2-F3	PO-PS F2-F4	WM-PS F3-F4
Step 1: configural invariance	340.90 (118)^∗^	0.92	0.09	104.90	0.07						
UK						0.47	0.46	0.18	0.58	0.61	0.45
SA						0.46	0.68	0.14	0.62	0.61	0.41
Step 2: metric invariance	405.15 (127)^∗^	0.90	0.10	151.15	0.10						
UK						0.48	0.48	0.18	0.59	0.60	0.45
SA						0.45	0.60	0.20	0.57	0.60	0.46
Step 3: strong factorial invariance	592.97 (136)^∗^	0.90	0.11	320.97	0.12						
						0.46	0.55	0.23	0.59	0.63	0.47

VC, Verbal Comprehension; PO, Perceptual Organization; WM, Working Memory; PS, Processing Speed; ^∗^*p* < 0.05.

The χ^2^ statistic is a commonly used index of goodness of fit for each model. It compares the degree to which the predicted covariances in the model differ from the observed covariances. A good fit is determined by small and non-significant χ^2^ values. Because this statistic is sensitive to variances in sample sizes, with large samples, as in the present study, even the best-fitting models frequently yield significant χ^2^ values (Kline, 1998).

Model adequacy was therefore evaluated using additional global fit indices, such as the Comparative Fit Index (CFI; Bentler, 1990), that are more sensitive to model specification than to sample size (Kline, 1998). Fit indices with values equal to or higher than 0.90 demonstrate a marginal fit, and values above 0.95 indicate a good fit. Further assessment of the extent to which the specified model approximates to the true model is the root mean square error of approximation (RMSEA) and standardized root mean square residual (SRMR). RMSEA and SRMR values below 0.05 indicate a good fit, values between 0.08 and 0.10 indicate a mediocre fit, and values above 0.10 indicate a poor fit (McDonald and Ho, 2002). We also included the AIC, a fit measure that takes into account the parsimony of models. Lower AIC values indicate a better fit (Bentler, 1990).

Step 1 was the baseline model in which no between-group restrictions were imposed. **Table 3** indicates that the baseline model fits reasonably well in terms of the CFI, AIC, and RMSEA. However, there is a difference in the factor loading between the UK and SA samples with respect to VC and WM. In Step 2, the factor loadings were restricted to be equal across both groups. As can be seen in **Table 3**, this restriction is accompanied by a significant increase in the chi-square value. The fit indices also worsen with this restriction. A chi-square difference test indicated a significant difference in fit between Step 1 and Step 2: χ^2^(9) = 64.26; *p*< 0.001. In Step 3, the measurement intercepts were restricted to be equal across both groups. As can be seen in **Table 3**, this restriction is accompanied by a significant increase in the chi-square value. The fit indices also worsen with this restriction. A chi-square difference test indicated a significant difference in fit between Step 2 and Step 3: χ^2^(9) = 187.82; *p*< 0.001. In this case, a step was accompanied by a clear deterioration in model fit and the particular restriction was rejected. Thus, the conclusion that can be drawn that there is some discrepancy in the model fit of a four-factor model of IQ between the British and South African samples and that the WAIS-III lacks measurement invariance across these samples.

### Exploratory Factor Analysis

Because of the poor fit on the CFA described above, and in order to investigate the factorial structure of the WAIS-III items cross-culturally, all 13 subtests were submitted to a principal components analysis (PCA), separately for the UK and SA students (**Table 4**).

**Table 4:** Principal components analyses.

	Factors			
	VCI (31.87%)	POI (14.33%)	WMI (9.53%)	PSI (8.15%)
**SA participants**
Information	0.839			
Vocabulary	0.816			
Similarities	0.763			
Comprehension	0.754			
Arithmetic	0.570		0.414	
Matrix Reasoning		0.771		
Picture Completion		0.765		
Block Design		0.596		
Picture Arrangement		0.495		
Digit Span			0.819	
Letter–Number Sequencing			0.776	
Digit-Symbol Coding				0.886
Symbol Search				0.735
**UK Participants**
	**POI (41.16%)**	**VCI (16.21%)**	**WMI (9.86%)**	

Picture Arrangement	0.830		
Symbol Search	0.765		
Digit-Symbol Coding	0.754		
Picture Completion	0.673		
Matrix Reasoning	0.655		
Block Design	0.632		
Vocabulary		0.850	
Similarities		0.843	
Information		0.810	
Comprehension		0.739	0.448	
Digit Span			0.875	
Letter–Number Sequencing			0.807	
Arithmetic		0.411	0.513	

VCI, Verbal Comprehension Index; POI, Perceptual Organization Index; WMI, Working Memory Index; PSI, Processing Speed Index.

The factor structure for the SA sample (factor loadings of >0.40) matched the specified WAIS-III indices for VCIs, POIs, WMIs, and PSIs. The only difference was that the Arithmetic subtest also loaded on the VCI.

In contrast, there was a different pattern for the UK sample, as eigenvalues greater than one indicated three factors (factor loadings of 0.40 or greater). The first factor included subtests from both the POI and PSI. The VCI and WMI were largely similar to those for the original WAIS-III. However, the Arithmetic subtest also loaded on the VCI and the Comprehension subtest also loaded on the WMI.

## Discussion

In the face of limited resources in third world countries, it is practical to avoid ‘re-inventing the wheel’ when it comes to measures of cognitive ability, but rather to research and adapt existing, psychometrically sound measures for use with local populations (Shuttleworth-Edwards, 1996). The WAIS is the most widely used and researched measure of adult intelligence, and it is thus practical to evaluate its use with populations quite different to the standardization samples, in this case multilingual, English second language speakers and from low socio-economic circumstances, yet with a sufficient degree of testwiseness and English proficiency. Of the factors known to influence IQ test performance, the SA groups were disadvantaged in several respects; while they had high self-reported proficiency in English, this was not their mother-tongue, they had received poor quality secondary schooling and came from low SES circumstances. When their performance was compared to a more aﬄuent, monolingual, UK group, it was found that the WAIS-III appears to lack measurement invariance across the two samples. An integrated interpretation of the statistical results pointed to three findings of interest. (1) There was no evidence of cultural biases in the Matrix Reasoning subtest or in the WM subtests that had minimal reliance on language. (2) All of the verbal and most non-verbal subtests, as well as the PS subtests, showed evidence of cross-cultural differences. (3) The SA and UK samples’ scores revealed different factor structures. Each of these findings is discussed in detail below.

The finding of equivalent performance between the SA and UK samples on the subtests of the WMI suggests that these may be the fairest measures of intellectual ability across culturally, linguistically, and socio-economically diverse groups. WM assessments draw on ability that is closely related to fluid intelligence, is not explicitly taught and is thus not knowledge-based. Since the procedures and stimuli used in WM tests are designed to be equally unfamiliar to all participants or draw on well-learned material, such as numbers and letters, they are less likely to confer obvious advantages or disadvantages to individuals from vastly differing backgrounds and are relatively uninfluenced by environmental factors such type and quality of education, SES and linguistic background (Alloway et al., 2004). This is particularly likely in the case of the LNS and Digit Span subtests of the WMI, which assess non-semantic, auditory aspects of WM. The LNS subtest of the WMI has been found to be the least vulnerable to socio-cultural effects in black Africans, with both advantaged and disadvantaged secondary and primary school education (Shuttleworth-Edwards et al., 2013). The third subtest in the WMI, Arithmetic, draws on several abilities (VC, WM, freedom from distractibility, numerical knowledge, and procedural knowledge) so that its consequent dual loading on both VCI and WMI for both UK and SA samples is not unexpected and has been demonstrated elsewhere (Grégoire, 2013). Its verbal content may mean that this subtest also holds some cultural biases and this may account for the differences in factor loadings between UK and SA samples on the WMI found in Step 1 of the MGCFA.

There is some debate regarding the extent to which WM tests are free of cultural biases (Ostrosky-Solis and Lozano, 2006). However, most cross-cultural differences pertain to articulatory rates in different languages for digits in the Digit Span task. Testing was conducted in English for both samples in the current study, thus eliminating this criticism. Cross-cultural differences in WM performance have been found with children from Zaire and Laos on the Kaufman Assessment Battery for Children (K-ABC; Boivin, 1991; Conant et al., 1999, 2003). It is possible that cross-cultural factors, as well as age of commencement of formal education and learning to read and write, may affect the developmental rate and organization of verbal and visuospatial components of WM development in children and greater stability may be evident in terms of these skills from early adulthood. This needs further investigation and may mean that WM assessments may only be suitable non-biased measures from young adulthood onward.

A similar explanation to that given for the WMI subtests would account for the absence of cross-cultural differences on the Matrix Reasoning subtest, which was added to the WAIS-III to ‘enhance the assessment of non-verbal, fluid reasoning’ (The Psychological Corporation, 1997, p. 6). The Matrix Reasoning subtest is modeled on the Raven’s Progressive Matrices, a classic measure of fluid reasoning and both tests are highly correlated (*r*= 0.80; Raven et al., 1988; Carpenter et al., 1990; The Psychological Corporation, 1997). The construct validity of the Matrix Reasoning subtest, however, has not been well-studied and the findings from the current study provide further evidence of its potential as a culture-fair measure.

Despite attempts to broaden the measurement of unbiased, fluid reasoning processes in the WAIS-III (The Psychological Corporation, 1997), the majority of its subtests appear to show cross-cultural effects. Given previous evidence, it was unsurprising that the verbal subtests showed the highest degree of cultural difference (Shuttleworth-Edwards et al., 2004; Razani et al., 2006). Cultural biases were also evident in two of the PO subtests (Block Design and Picture Completion), as well as Picture Arrangement. This supports the view that non-verbal tests are not necessarily culturally fairer than verbal ones (Rosselli and Ardila, 2003; Shuttleworth-Edwards et al., 2013). Past cross-cultural research on the Block Design subtest has yielded mixed results. Some identified it as discriminating against individuals from non-Western cultures and/or deprived educational backgrounds (Ardila and Moreno, 2001; Shuttleworth-Edwards et al., 2004, 2013). Other studies have found no significant differences between Anglo-Americans and fluent English-speaking individuals from ethnically diverse (Hispanic, Asian, and Middle-Eastern) backgrounds on Block Design and Matrix Reasoning (Razani et al., 2006). These differences may be due to differing levels of acculturation in the various samples. Most differences between the UK and SA samples in the current study were found on verbal and non-verbal measures that draw on acquired knowledge and learned problem solving strategies.

The significant difference between the SA and UK samples in terms of PS, in favor of the SA sample was surprising given a body of research indicating that PS is an area where individuals of African descent underperform relative to Caucasians and Hispanics from Westernized, urbanized countries. Reduced PS by individuals of African origin is proposed to be due to a tendency to value careful deliberation and accuracy over speed (Nell, 2000; Heaton et al., 2003; Shuttleworth-Edwards et al., 2004). The reasons for the contrary finding in the current study are most likely a result of multiple interacting factors, which may include sampling and socio-cultural changes in SA over the last 20 years, including the Flynn (1984, 1987, 2009) effect.

In terms of sampling bias, both samples in this study comprised student volunteers. This yielded some differences between the two countries. Although the students were all undergraduates, there was much greater variance in the ages of the UK sample (*M*= 27.18 years; SD = 8.74 years), while the SA sample was fairly homogenous in terms of age (*M*= 20.79; SD = 1.56 years). Increased age is associated with a decrease in PS and although age-adjusted standard scores were used in the analyses, this may not completely exclude all variance associated with age (Lezak et al., 2004).

Another issue that may have influenced the results on the PSI are socio-cultural ones resulting from a changed SA society since the ending of apartheid 20 years ago. As a consequence of these changes, the ‘born free’ generation (children born after the end of apartheid, as is the case in the current SA sample) are less different to Western populations than previous generations of black South Africans. The ‘born free’ generation has witnessed dramatic changes in comparison to their parents. There has been increased access to technology, with all students possessing mobile phones, most which allow internet access and consequent acculturation; the state has provided access to child and health care, family planning and nutrition; social grants have expanded from 2.7 million people in 1994 to 16 million in 2014; there has been an emphasis on, and investment in, education and the school curriculum has been changed from one that focused on rote repetition and obedience to authority, to a focus on the scientific method and active problem solving (although there are still inequities in the quality of education provided by the different education systems; Maloka, 2013). Other positive changes include electrification of homes – in 1994, 59.5% of SA households had access to electricity. Statistics South Africa (2012), this had risen to 85.3%. The proportion of formal households has increased from 63.1% in 1996, to 77.6% in 2011 and those residing in shacks or informal dwellings has decreased over the same period from 16.2 to 13.6% (Statistics South Africa, 2011). These changes altered not only the quality of life for a large proportion of black South Africans, but have also led to a change in self-esteem in the black community – from being marginalized, to being a valued member of society.

Several of the socio-cultural factors mentioned above are associated with the Flynn effect, which are reported to be strongest on fluid measures (e.g., Matrix Reasoning). Since WM and PS are strongly related to fluid ability, concomitant influences could also be expected in these areas (Weiss, 2010). If the current SA data are compared to that of Shuttleworth-Edwards et al. (2004, 2013) for black Africans with an ex-DET education and 15+ years of education (*n*= 12), published 10 years ago, the data are within 1 standard deviation of each other, and do not differ significantly in terms of Index and IQ scores. Nonetheless, the slightly higher PSI obtained in the current study relative to that of Shuttleworth-Edwards et al. (2004, 2013) could arguably be reflective of Flynn effects combined with the better life circumstances and opportunities for present day black South Africans.

Returning to the PSI comparisons between the SA and UK groups in the current study, these indicate functioning within the average range for the South African sample, and performance that is in the low average range for the UK group, which is unusual, particularly for a university population. In an attempt to better understand these differences, the factor structure of the WAIS-III scores for each country was analyzed, since a comparison of means is based on the assumption that the meaning of the items and the subtests is similar in the groups being compared, which would imply that the factor structure is also similar in both groups. The factor structure of the WAIS-III was not equivalent for the UK and SA samples. A four factor structure provided the best fit for the SA sample, following the four indices of the WAIS-III, although Arithmetic loaded on both WMI and VCI. Interestingly, a four factor structure could not be replicated with the UK sample. Instead, a three factor model provided the best fit, where the PSI subtests loaded together with those measuring non-verbal, PO and reasoning skills.

This finding suggests that the UK sample utilized similar cognitive processes to complete the non-verbal PSI and POI subtests, which were possibly a combination of accuracy and speed since some of the POI subtests are timed. In support of this, European individuals (from Germany, France, UK, Finland, and Spain) have been found to focus on accuracy over speed on the WAIS-III in comparison to US individuals (Roivainen, 2010). Unlike the UK sample, the SA group appeared to distinguish between the POI and PSI subtests, drawing on different skills for each index. Thus, the discrepancy in performance on the PSI is explicable in terms of the differing factor structures in the two groups. The PS performance of the SA group may also account for their performance on the WM tests, as faster processing allows for greater mental rehearsal, faster execution of ongoing tasks and greater synchronization of task components in WM (Nettelbeck and Burns, 2010).

In conclusion, the findings from this study add to existing evidence that the majority of the subtests in the WAIS-III hold cross-cultural biases. These are most evident in tasks which tap crystallized, long-term learning, irrespective of whether the format is verbal or non-verbal. This challenges the view that visuo-spatial and non-verbal tests tend to be culturally fairer than verbal ones (Rosselli and Ardila, 2003). Subtests tapping PS also appear to hold biases. Differences on the latter subtests may reflect cultural and/or experiential background. Of value for theory and practice, those subtests tapping WM and fluid reasoning (Matrix Reasoning) showed no cross-cultural effects. Consequently, one of the most effective ways of tapping universal cognitive functions may be via WM and this warrants further investigation. Identification of tests that do not favor individuals from Eurocentric and favorable SES circumstances with advantaged educational backgrounds is valuable in providing direction for the development of culture fair tests. Given the findings of the current study and evidence of a strong relationship between WM and educational outcomes (Cowan and Alloway, 2008; Alloway and Alloway, 2010), measures of WM offer promise in the search for fairer assessment practices with individuals from diverse backgrounds.

## Conflict of Interest Statement

The authors declare that the research was conducted in the absence of any commercial or financial relationships that could be construed as a potential conflict of interest.

## References

[B1] Alloway T. P. Alloway R. G. (2010). Investigating the predictive roles of working memory and IQ in academic attainment. *J. Exp. Child Psychol.* 106 20–29 10.1016/j.jecp.2009.11.003 20018296

[B2] Alloway T. P. Gathercole S. E. Willis C. Adams A. M. (2004). A structural analysis of working memory and related cognitive skills in early childhood. *J. Exp. Child Psychol.* 87 85–106 10.1016/j.jecp.2003.10.002 14757066

[B3] Ardila A. Moreno S. (2001). Neuropsychological test performance in Aruaco Indians: an exploratory study. *J. Int. Neuropsychol. Soc.* 7 510–515 10.1007/s10903-011-9443-z 11396553

[B4] Barber N. (2005). Educational and ecological correlates of IQ: a cross-national investigation. *Intelligence* 33 273–284 10.1016/j.intell.2005.01.001

[B5] Bentler P. M. (1990). Comparative fit indexes in structural models. *Psychol. Bull.* 107 238–246 10.1007/BF02294170 2320703

[B6] Boivin M. J. (1991). The effect of culture on a visual-spatial memory task. *J. Gen. Psychol.* 118 327–334 10.1007/978-1-4614-6834-9 1813596

[B7] Bollen K. A. (1989). *Structural Equations With Latent Variables*. Oxford: John Wiley and Sons. 10.1002/9781118619179

[B8] Boone K. B. Victor T. L. Wen J. Razani J. Ponton M. (2007). The association between neuropsychological scores and ethnicity, language, and acculturation variables in a large patient population. *Arch. Clin. Neuropsychol.* 22 355–365 10.1016/j.acn.2007.01.010 17320344

[B9] Byrd D. A. Miller S. Reilly J. Weber S. Wall T. L. Heaton R. K. (2006). Early environmental factors, ethnicity, and adult cognitive test performance. *Clin. Neuropsychol.* 20 243–260 10.1080/13854040590947489 16690545

[B10] Campbell T. Dollaghan C. Needleman H. Janosky J. (1997). Reducing bias in language assessment: processing dependent measures. *J. Speech Lang. Hear. Res.* 40 519–525 10.1044/jslhr.4003.519 9210111

[B11] Carpenter P. A. Just M. A. Shell P. (1990). What one intelligence test measures: a theoretical account of the processing in the Raven Progressive Matrices Test. *Psychol. Rev.* 97 404–431 10.1037/0033-295X.97.3.404 2381998

[B12] Claassen N. C. W. Krynauw A. H. Paterson H. Wa ga Mathe M. (2001). *A Standardisation of the WAIS-III for English-Speaking South Africans*. Pretoria: Human Sciences Research Council.

[B13] Conant L. L. Fastenau P. S. Giordani B. Boivin M. J. Chounramany C. Xaisida S. (2003). Environmental influences on primary memory development: a cross-cultural study of memory span in Lao and American children. *J. Clin. Exp. Neuropsychol.* 25 1102–1116 10.1076/jcen.25.8.1102.16722 14566584

[B14] Conant L. L. Fastenau P. S. Giordani B. Boivin M. J. Opel B. Dia Nseyila D. (1999). Modality specificity of memory span tasks among Zaïrean children: a developmental perspective. *J. Clin. Exp. Neuropsychol.* 21 375–384 10.1076/jcen.21.3.375.912 10474176

[B15] Cowan N. Alloway T. P. (2008). “The development of working memory in childhood,” in *Development of Memory in Infancy and Childhood*, 2nd Edn, eds M. Courage and N. Cowan (Hove: Psychology Press), 303–342.

[B16] Department of Education. (2008). *South African Schools Act (84 of 1996). National Norms and Standards for School Funding. Government Gazette No.* 30332 Notice no. 883 DoE: Pretoria.

[B17] Dolan C. V. Colom R. Abad F. J. Wicherts J. M. Hessen D. J. van der Sluis S. (2006). Multi-group covariance and mean structure modeling of the relationship between the WAIS-III common factors and sex and educational attainment in Spain. *Intelligence* 34 193–210 10.1016/j.intell.2005.09.003

[B18] Dolan C. V. Roorda W. Wicherts J. M. (2004). Two failures of Spearman’s hypothesis: the GAT-B in Holland and the JAT in South Africa. *Intelligence* 32 155–173 10.1016/j.intell.2003.09.001

[B19] Dugbartey A. T. Sanchez P. N. Rosenbaum J. G. Mahurin R. K. Davis J. M. Townes B. D. (1999). WAIS-III Matrix reasoning test performance in a mixed clinical sample. *Clin. Neuropsychol.* 13 396–404 10.1076/1385-4046(199911)13:04;1-Y;FT396 10806451

[B20] Fleisch B. (2008). *Primary Education in Crisis: Why South African Children underperform in Reading and Mathematics*. Cape Town: Juta and Co. Ltd.

[B21] Flynn J. R. (1984). The mean IQ of Americans: massive gains 1932 to 1978. *Psychol. Bull.* 95 29–51 10.1037/0033-2909.95.1.29

[B22] Flynn J. R. (1987). Massive IQ gains in 14 nations: what IQ tests really measure. *Psychol. Bull.* 101 171–191 10.1037/0033-2909.101.2.171

[B23] Flynn J. R. (2007). *What is Intelligence? Beyond the Flynn Effect*. Cambridge: Cambridge University Press. 10.1017/CBO9780511605253

[B24] Flynn J. R. (2009). The WAIS-III and WAIS-IV: daubert motions favour the certainly false over the approximately true. *Appl. Neuropsychol.* 16 98–104 10.1080/0908428090286436 19430991

[B25] Foxcroft C. D. Aston S. (2006). Critically examining language bias in the South African adaptation of the WAIS-III. *S. Afr. J. Ind. Psychol.* 32 97–102.

[B26] Gonzales R. R. Roll S. (1985). Relationship between acculturation, cognitive style, and intelligence. A cross-sectional study. *J. Cross Cult. Psychol.* 16 190–205 10.1177/0022002185016002004

[B27] Grégoire J. (2004). Factor structure of the French version of the Wechsler Adult intelligence scale-III. *Educ. Psychol. Meas.* 64 463–474 10.1177/0013164403258452

[B28] Grégoire J. (2013). Measuring components of intelligence: mission impossible? *J. Psychoed. Assess.* 31 138–147 10.1177/0734282913478034

[B29] Harris J. G. Tulsky D. Schultheis M. T. (2003). “Assessment of the non-native English speaker: assimilating history and research findings to guide clinical practice,” in *Clinical Interpretation of the WAIS-III and WMS-III*, eds D.S. Tulsky, D.H. Saklofske, G. J. Chelune, R. K. Heaton, and R. J. Ivnik (San Diego, CA: Academic Press), 343–390.

[B30] Heaton R. K. Taylor M. J. Manly J. (2003). “Demographic effects and use of demographically corrected norms with the WAIS-III and WMS-III”, in *Clinical Interpretation of the WAIS-III and WMS-III*, eds D. S. Tulsky, D. H. Saklofske, G. J. Chelune, R. K. Heaton, and R. J. Ivnik (San Diego, CA: Academic Press), 183–219 10.1016/B978-012703570-3/50010-9

[B31] Helms J. E. (1992). Why is there no study of cultural equivalence in standardized cognitive ability testing? *Am. Psychol.* 47 1083–1101 10.1037/0003-066X.47.9.1083

[B32] Kaufman A. S. (1994). *Intelligent Testing With the WISC-III*. New York: John Wiley and Sons.

[B33] Kaufman A. S. McLean J. E. Reynolds C. R. (1988). Sex, residence, region, and education differences on the 11 WAIS-R subtests. *J. Clin. Psychol.* 44 213–248 10.1002/1097-4679(198803)44 3360941

[B34] Kaufman A. S. McLean J. E. Reynolds C. R. (1991). Analysis of WAIS-R factor patterns by sex and race. *J. Clin. Psychol.* 47 548–557 10.1002/1097-4679(199107)47:4<548::AID-JCLP2270470413>3.0.CO;2-# 1939700

[B35] Kennepohl S. Shore D. Nabors N. Hanks R. (2004). African American acculturation and neuropsychological test performance following traumatic brain injury. *J. Int. Neuropsychol. Soc.* 10 566–577 10.1017/S1355617704104128 15327735

[B36] Kessels R. P. Wingbermuhle P. A. (2001). The Dutch version of the WAIS-III: a measure of intelligence and cognitive functioning. *Ned. Tijdschr. Psychol. Grens*. 56 227–230.

[B37] Kline R. B. (1998). *Principles and Practice of Structural Equation Modelling*. New York: The Guilford Press.

[B38] Lezak M. D. Howieson D. B. Loring D. W. (2004). *Neuropychological Assessment,* 4th Edn. Oxford: Oxford University Press.

[B39] Maloka E. (2013). *The Fruits of Freedom South African History Online*. http://www.sahistory.org.za/archive/chapter-4-fruits-freedom [accessed April 15, 2014].

[B40] Manly J. J. Byrd D. Touradji P. Sanchez D. Stern Y. (2004). Literacy and cognitive change among ethnically diverse elders. *Int. J. Psychol.* 39 47–60 10.1080/00207590344000286

[B41] Manly J. J. Miller S. W. Heaton R. K. Byrd D. Reilly J. Velasquez R. J. (1998). The effect of African-American acculturation on neuropsychological test performance in normal and HIV-positive individuals. *J. Int. Neuropsychol. Soc.* 4 291–302. 9623004

[B42] Marcopulos B. A. McLain C. A. Giuliano A. J. (1997). Cognitive impairment or inadequate norms? A study of healthy, rural, older adults with limited education. *Clin. Neuropsychol.* 11 111–131 10.1080/13854049708407040

[B43] McDonald R. P. Ho M. R. (2002). Principles and practice in reporting structural equation analyses. *Psychol. Methods* 7 64–82 10.1037/1082-989X.7.1.64 11928891

[B44] Milfont T. L. Fischer R. (2010). Testing measurement invariance across groups: applications in cross-cultural research. *Int. J. Psychol. Res.* 3 111–121 10.1177/2158244013515686

[B45] Nell V. (2000). *Cross-cultural Neuropsychological Assessment: Theory and Practice*. New Jersey: Lawrence Erlbaum Associates.

[B46] Nettelbeck T. Burns N. R. (2010). Processing speed, working memory and reasoning ability from childhood to old age. *Pers. Individ. Dif.* 48 379–384 10.1016/j.paid.2009.10.032

[B47] Ostrosky-Solis F. Lozano A. (2006). Digit span: effect of education and culture. *Int. J. Psychol.* 41 333–341 10.1080/00207590500345724

[B48] Perry J. L. Satiani A. Henze K. T. Mascher J. Helms K. T. (2008). Why is there *Still* no study of cultural equivalence in standardized cognitive ability tests? *J. Multicult. Counsel. Dev.* 36 155–167 10.1002/j.2161-1912.2008.tb00079.x

[B49] Raven J. C. Court J. H. Raven J. (1988). Manual for Raven’s Progressive Matrices and Vocabulary Scales. London: H. K. Lewis.

[B50] Razani J. Burciaga J. Madore M. Wong J. (2007). Effects of acculturation on tests of attention and information processing in an ethnically diverse group. *Arch. Clin. Neuropsychol.* 22 331–341 10.1016/j.acn.2007.01.008 17298874

[B51] Razani J. Murcia G. Tabares J. Wong J. (2006). The effects of culture on WASI test performance in ethnically diverse individuals. *Clin. Neuropsychol.* 21 776–788 10.1080/13854040701437481 17676543

[B52] Roivainen E. (2010). European and American WAIS III norms: cross-national differences in performance subtest scores. *Intelligence* 38 187–192 10.1016/j.intell.2009.10.001 31587302

[B53] Rosselli M. Ardila A. (2003). The impact of culture and education on nonverbal neuropsychological measurements: a critical review. *Brain Cogn.* 52 326–333 10.1016/S0278-2626(03)00170-2 12907177

[B54] Shores E. A. Carstairs J. R. (2000). The macquarie university neuropsychological normative study (MUNNS): australian norms for the WAIS-R and WMS-R. *Aust. Psychol.* 35 41–59 10.1080/00050060008257467

[B55] Shuttleworth-Edwards A. B. (1996). On not re-inventing the wheel: a clinical perspective on culturally relevant test usage in South Africa. *S. Afr. J. Psychol.* 26 96–102 10.1177/008124639602600205

[B56] Shuttleworth-Edwards A. B. Gaylard E. K. Radloff S. E. (2013). “WAIS-III test performance in the South African context: extension of a prior cross-cultural normative database,” in *Psychological Assessment in South Africa: Research and Applications*, eds S. Laher and K. Cockcroft (Johannesburg: Wits University Press), 17–32.

[B57] Shuttleworth-Edwards A. B. Kemp R. D. Rust A. L. Muirhead J. G. L. Hartman N. P. Radloff S. E. (2004). Cross-cultural effects on IQ test performance: a review and preliminary normative indications on WAIS-III test performance. *J. Clin. Exp. Neuropsychol.* 26 903–920 10.1080/13803390490510824 15742541

[B58] Statistics South Africa. (2011). *Social Profile of Vulnerable Groups in South Africa 2002–2011.* http://www.statssa.gov.za/publications/Report-03-19-00/Report-03-19-002011.pdf [accessed April 21, 2014].

[B59] Statistics South Africa. (2012). *Annual Financial Statistics (AFS), 2012, November 26, 2013.* https://www.statssa.gov.za/publications/statsdownload.asp?PPN=P0021&SCH=5692 [accessed April 21, 2014].

[B60] te Nijenhuis J. (2013). Flynn effect, group differences, and *g* loadings. *Pers. Individ. Dif.* 55 224–228 10.1016/j.paid.2011.12.023

[B61] te Nijenhuis J. Murphy R. van Eeden R. (2011). The Flynn effect in South Africa. *Intelligence* 39 456–467 10.1016/j.intell.2011.08.003

[B62] The Psychological Corporation. (1997). *Wechsler Adult Intelligence Scale – III technical manual*. San Antonio, TX: The Psychological Corporation.

[B63] Turkheimer E. Haley A. Waldron M. D’Onofrio B. Gottesman I. I. (2003). Socioeconomic status modifies heritability of IQ in young children. *Psychol. Sci.* 14 623–628 10.1046/j.0956-7976.2003.psci-1475.x 14629696

[B64] Walker A. J. Batchelor J. Shores A. (2009). Effects of education and cultural background on performance on WAIS-III, WMS-III, WAIS-R and WMS-R measures: systematic review. *Aust. Psychol.* 44 216–223 10.1080/00050060902833469

[B65] Wechsler D. (1997). Wechsler Adult Intelligence Scale-Third Edition. London: The Psychological Corporation Limited.

[B66] Weiss L. G. (2010). Considerations on the Flynn Effect. *J. Psychoed. Assess.* 28 482–493 10.1177/0734282910373572

[B67] Wicherts J. M. Dolan C. V. (2010). Measurement invariance in confirmatory factor analysis: an illustration using IQ test performance of minorities. *Educ. Meas. Issues Pract*. 29 39–47 10.1111/j.1745-3992.2010.00182.x

